# Choledocholithiasis as a risk factor for cholangiocarcinoma: a nationwide retrospective cohort study

**DOI:** 10.1186/s12876-025-03746-w

**Published:** 2025-03-05

**Authors:** Jaihwan Kim, Yoon Suk Lee, Jong-Chan Lee, Jin-Hyeok Hwang

**Affiliations:** 1 Department of Internal Medicine, Seoul National University Bundang Hospital, Seoul National University College of Medicine, Seongnam, South Korea; 2 Department of Internal Medicine, Ilsan Paik Hospital, Inje University College of Medicine, 170, Juhwa-ro, Ilsanseo-gu, Goyang, South Korea

**Keywords:** Choledocholithiasis, Cholangiocarcinoma, Risk factors, Cohort studies

## Abstract

**Background:**

Choledocholithiasis has been reported to be associated with the occurrence of cholangiocarcinoma (CCA); however, the association has not yet been sufficiently demonstrated. This study aimed to evaluate the association between choledocholithiasis (common bile duct stones) and CCA.

**Methods:**

This nationwide retrospective cohort study used the Health Insurance Review and Assessment database of individuals diagnosed with choledocholithiasis between 2008 and 2009 in South Korea. Individuals were stratified by age, and CCA was categorized into extrahepatic CCA (ECA) and intrahepatic CCA (ICA). The standardized incidence ratio (SIR) was calculated to compare CCA incidence between patients with choledocholithiasis and the general population.

**Results:**

The study enrolled 20,808 patients with choledocholithiasis (52.35% men and 47.65% women; male-to-female ratio: 1.09:1). Over a 10-year follow-up period, CCA occurred in 548 (2.64%) patients, comprising 238 (1.14%) ECA cases and 310 (1.48%) ICA cases. The SIR was 25.23 (95% confidence interval [CI]: 21.98–28.85) for ECA and 24.64 (95% CI: 21.87–27.73) for ICA. Statistical significance persisted even after excluding cases within the first 2 years from the index date, with an SIR of 18.63 (95% CI: 16.23–21.28) for ICA and 12.73 (95% CI: 10.50–15.30) for ECA. The SIRs peaked in patients diagnosed with choledocholithiasis at the age of 70–79 years (SIR 16.61, 95% CI: 11.83–22.69) for ECA and 60–69 years (SIR 29.27, 95% CI: 23.53–36.03) for ICA.

**Conclusion:**

Our study demonstrated a significant association between choledocholithiasis and cholangiocarcinoma, particularly those in their 70s for ECA and 60s for ICA. However, causation cannot be established due to the retrospective design.

**Supplementary Information:**

The online version contains supplementary material available at 10.1186/s12876-025-03746-w.

## Introduction

Cholangiocarcinoma (CCA) is a group of cancers that originate from any portion of the bile duct epithelium [1]. It accounts for 3% of gastrointestinal tumors and approximately 10–25% of all hepatobiliary malignancies [2, 3]. Choledocholithiasis has recently been suggested as a risk factor for CCA. Two large Surveillance, Epidemiology, and End Results (SEER)-Medicare studies revealed a strong positive association between CCA and choledocholithiasis, with risk estimates ranging 4–64 [4, 5]. Consistently, a population-based study from Denmark also demonstrated a significant association between choledocholithiasis and CCA [6]. However, these studies used retrospective designs because conducting prospective cohort studies is difficult, given the low incidence of CCA and the relatively long duration for the subsequent development duration of CCA [4–9], requiring further assessment of the association.

Furthermore, the higher incidence of CCA in South Korea than in Western countries could provide a better environment to investigate the association. Therefore, we conducted a nationwide population-based retrospective cohort study to investigate the association between choledocholithiasis and CCA using the Health Insurance Review and Assessment Service (HIRA) database in South Korea.

## Patients and methods

### Data source

The National Health Insurance Service (NHIS) and the HIRA are national health insurance systems in South Korea that prospectively compile all claim records for medical services [10, 11] and have been used in cohort studies [12–17]. The NHIS, which is a national institution for managing national health insurance in South Korea, provides healthcare services to residents, covering most medical conditions except for cosmetic treatments [18]. Each claim for medical expense reimbursement is reviewed for appropriateness by the HIRA, which is a government agency under the Ministry of Health and Welfare. Consequently, claims data for medical services covered by the NHIS have been prospectively deposited in the HIRA database, which is available for academic purposes since 2010, upon receiving approval from the HIRA review board or qualified researchers. This study utilized three datasets from the HIRA database: one for general information containing demographic data, such as sex, age, and indicators for inpatient and outpatient services; a second for specific information on provided services, including the serial number of claims for medical reimbursement, the items and clauses of the claims for specific treatments (such as drugs administered, types of surgery or endoscopic procedures); and a third for diagnostic information corresponding to each claim. The key codes for each dataset were unique and connected to the datasets.

### Ethics declarations

Approval from the Institutional Review Board and Ethics Committee of Seoul National University Bundang Hospital (approval number: X-1907-550-906) was obtained. However, the requirement for a consent declaration from participants was waived for this study, as personally identifiable patient information was neither collected nor recorded. All procedures were performed in accordance with the Declaration of Helsinki.

### Population-based cohort

We employed specific operational definitions, utilizing diagnostic and procedural codes exclusively for in-hospital medical services (excluding outpatient services) to establish a patient cohort with choledocholithiasis. This included individuals admitted to medical institutions with the Korean Standard Classification of Disease (KCD) diagnostic codes for choledocholithiasis (K80.50, K80.51, K80.30, K80.31, K80.40, and K80.41) and procedure registry codes for endoscopic biliary stone removal (Q7764 and Q7765), endoscopic biliary drainage (Q7762), and endoscopic sphincterotomy (Q7761). Supplementary Table 1 summarizes the KCD codes used in this study (https://www.kcdcode.kr/browse/main). We extracted individual data from January 1, 2007, to December 31, 2009, using the sixth version of the KCD in the HIRA database during this period. To establish a clear washout period, individuals with recurrent choledocholithiasis diagnosed with KCD codes between January 1, 2007, and December 31, 2007, were excluded. Furthermore, individuals claiming any gastrointestinal malignancies, including stomach cancer, colon cancer, pancreatic cancer, CCA (intrahepatic CCA [ICA] and extrahepatic CCA [ECA]), hepatocellular carcinoma, and gallbladder cancer, were excluded from the study, regardless of the timing, throughout the three-year study inclusion period.

This choledocholithiasis cohort was monitored until the occurrence of CCA, which was defined as new claims for medical services with KCD diagnostic codes of CCA (C22.1 for ICA and C24.0 for ECA) subsequent to the choledocholithiasis diagnosis. All claims with the abovementioned KCD codes are documented as principal or first secondary diagnoses. This study design, employing operational definitions, has previously demonstrated efficacy in population-based cohort studies utilizing the HIRA database in South Korea [13, 19]. Consequently, the incidence of CCA can be assessed for almost the entire Korean population, enabling the execution of a nationwide population-based cohort study.

### Statistics

Crude incidence reflects the number of patients newly requesting reimbursement based on CCA diagnostic codes. The incidence of subsequent CCA following choledocholithiasis diagnosis was compared with that of the general population (100,000 individuals) to estimate standardized incidence ratios (SIRs). General population incidence rates were obtained from the Korean Cancer Registry at the National Cancer Center, which was used to compute the expected numbers [20]. The 2012 Korean population estimate from the Korean Statistical Information Service (https://kosis.kr/eng) served as the standard population for calculations. The 95% confidence interval (CI) for SIRs was determined, assuming a Poisson distribution for observed cancer cases. Cumulative incidence curves displayed the predicted cancer risk in patients with cancer in our study. We stratified the choledocholithiasis cohort based on age at the index date of choledocholithiasis to evaluate the effect of age on incidence rates. Furthermore, given that the median survival of patients with CCA in South Korea was reported to be about 8 months (range: 2.8–23.3) for ICA and 16.6 months (range: 5.3–41.9) for ECA [20], the incidence of CCA was investigated between the first two years and the subsequent years; the SIRs were calculated after excluding the cases of CCA that developed within the first 2 years following the choledocholithiasis diagnosis in order to mitigate the possibility of including cases of choledocholithiasis secondary to tumor-induced cholestasis. The Chi-square test and independent t-test were used to compare the frequencies of categorical and continuous values, respectively. Kaplan–Meier survival analysis was performed to generate survival plots. Statistical significance was defined as a *P-*value of < 0.05. All analyses were performed using SAS enterprise software (version 9.4; SAS Institute, Cary, NC, USA) to draw plots.

## Results

### Study population

This study enrolled 20,808 patients diagnosed with choledocholithiasis between January 2008 and December 2009. The median age of the patients was 65 years [interquartile range, 52–74 years]. The male-to-female sex ratio was 1.09, comprising 10,894 men and 9,914 women. The age distribution of the enrolled patients was as follows: 4,340 (20.86%) were < 50 years, 3,602 (17.31%) were 50–59 years, 5,221 (25.09%) were 60–69 years, 5,336 (35.64%) were 70–79 years, and 2,309 (11.10%) were ≥ 80 years. The age group of 60–69 years had the highest number of men, whereas the age group of 70–79 years had the highest number of women (Table 1).

**Table 1:** Study population

	Total number of patients	Male	Female	*p*-value
	20,808	10,894 (52.35%)	9,914 (47.65%)	
Age, median (interquartile range)	65 (52–74)	64 (53–72)	66 (52–75)	< 0.001
Age stratified				< 0.001
< 50	4,340 (20.86%)	2,148 (19.72%)	2,192 (22.11%)	
50–59	3,602 (17.31%)	2,048 (18.80%)	1,554 (15.67%)	
60–69	5,221 (25.09%)	3,037 (27.88%)	2,184 (22.03%)	
70–79	5,336 (25.64%)	2711 (24.89%)	2625 (26.48%)	
≥ 80	2,309 (11.10%)	950 (8.72%)	1359 (13.71%)	

### Crude incidence of CCA after choledocholithiasis

CCA cases were reported in 548 (2.64%) of the 20,808 patients diagnosed with choledocholithiasis over a 10-year follow-up period. Among these patients, 238 (1.14%) had ECA, and 310 (1.48%) had ICA. After stratifying patients according to age at choledocholithiasis diagnosis into the < 50 year, 50–59 year, 60–69 year, 70–79 year, and ≥ 80 year age groups, ECA was reported in 12 (0.28%) of 4,340 patients, 39 (1.08%) of 3,602 patients, 77 (1.47%) of 5,221 patients, 84 (1.57%) of 5,336 patients, and 26 (1.14%) of 2,309 patients, respectively, indicating the highest risk of ECA in the 70–79 year age group; for ICA, the reported cases were 16 (0.37%), 53 (1.47%), 118 (2.26%), 94 (1.76%), and 29 (1.26%) patients, respectively, indicating the highest risk of ICA in the 60–69 year age group (Table 2).

**Table 2:** Crude incidence of cholangiocarcinoma within 0–2 years and 2 years after the choledocholithiasis diagnosis

		All cholangiocarcinoma (*n* = 548)		Extrahepatic cholangiocarcinoma (*n* = 238)		Intrahepatic cholangiocarcinoma (*n* = 310)	
Age at enrollment	Total No. (*n* = 20,808)	Within 0–2 years (*n* = 194)	After ≥ 2 years (*n* = 354)	Within 0–2 years (*n* = 118)	After ≥ 2 years (*n* = 120)	Within 0–2 years (*n* = 76)	After ≥ 2 years (*n* = 234)
< 50	4,340	14 (0.32%)	14 (0.32%)	7 (0.16%)	5 (1.15%)	7 (0.16%)	9 (0.21%)
50–59	3,602	24 (0.67%)	68 (1.89%)	16 (0.44%)	23 (0.64%)	8 (0.22%)	45 (1.25%)
60–69	5,221	64 (1.22%)	131 (2.51%)	38 (0.73%)	39 (0.75%)	26 (0.49%)	92 (1.76%)
70–79	5,336	71 (1.33%)	107(2.01%)	44 (0.82%)	40 (0.75%)	27 (0.51%)	67 (1,25%)
≥ 80	2,309	21 (0.91%)	34 (1.47%)	13 (0.56%)	13 (0.56%)	8 (0.35%)	21 (0.91%)

### Cumulative incidence and SIR of CCA

**Fig. 1:**
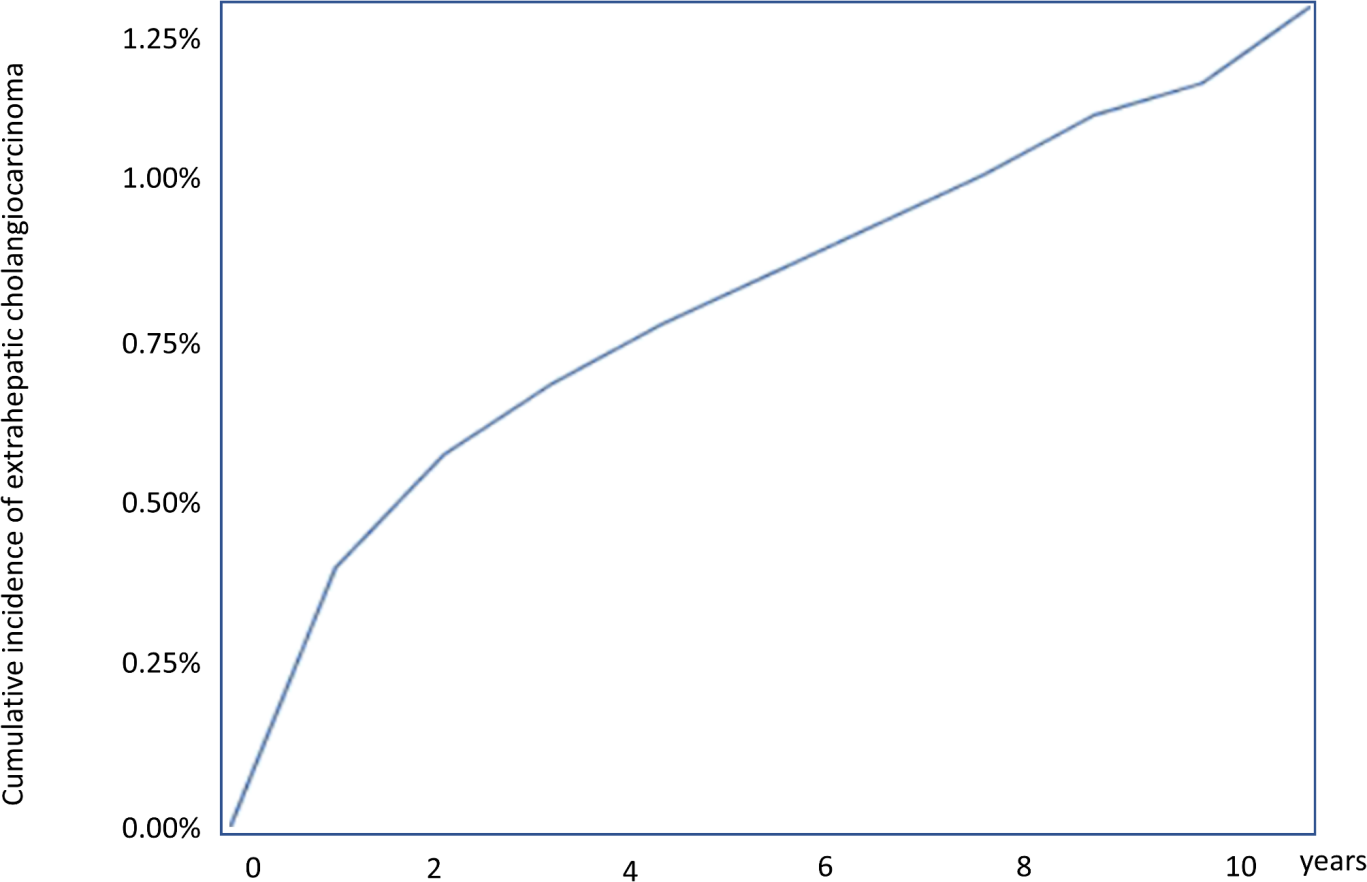
Cumulative incidence of extrahepatic cholangiocarcinoma in patients with choledocholithiasis. The incidence of extrahepatic cholangiocarcinoma increased progressively over time with a relatively rapid slope observed during the first 2 years

**Fig. 2:**
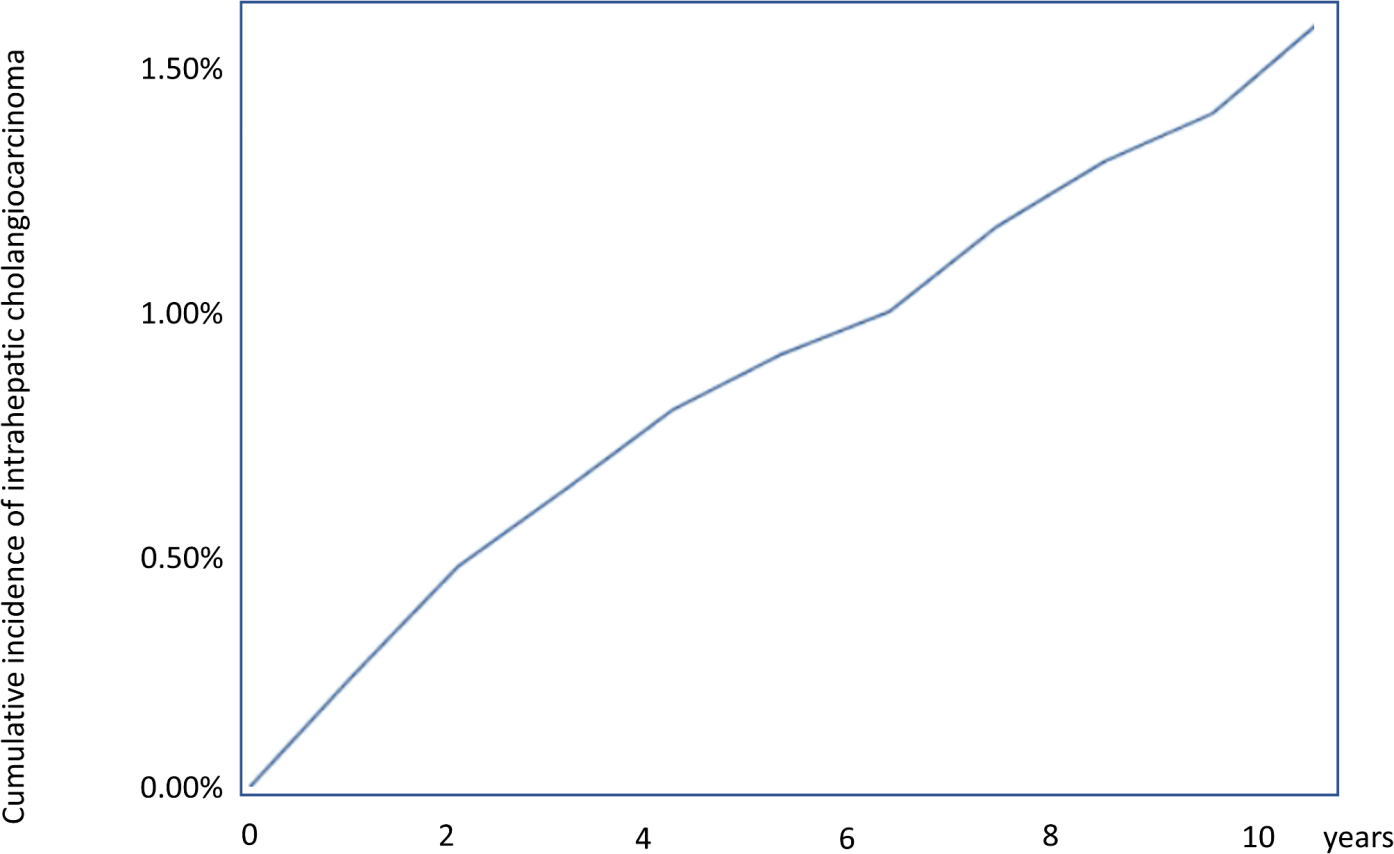
Cumulative incidence of intrahepatic cholangiocarcinoma in patients with choledocholithiasis. The incidence of intrahepatic cholangiocarcinoma rises progressively over time

Almost half of the patients (118/238 [49.57%]) received the diagnosis of ECA within the first 2 years after choledocholithiasis diagnosis, exhibiting a relatively higher cumulative incidence rate within this initial period compared to more than 2 years following the choledocholithiasis diagnosis (Fig. 1). Conversely, ICA was diagnosed in only one-fourth of the patients (76/310 [24.5%]) within the first 2 years, with a steady cumulative incidence rate over time following the choledocholithiasis diagnosis (Fig. 2).

The SIRs of CCA after choledocholithiasis were 25.23 (95% CI: 21.98–28.85) and 24.64 (95% CI: 21.87–27.73) for ECA and ICA, respectively. Furthermore, we excluded cases that CCA was diagnosed within the first 2 years following the choledocholithiasis diagnosis, considering that choledocholithiasis could manifest as an initial presentation of occult co-existing CCA. After this exclusion, SIR was 18.63 (95% CI: 16.23–21.28) for ICA and 12.73 (95% CI: 10.50–15.30) for ECA. The SIRs peaked in individuals aged 70–79 years (SIR: 16.61, 95% CI: 11.83–22.69) for ECA and 60–69 years (SIR: 29.27, 95% CI: 23.53–36.03) for ICA (Table 3).

**Table 3:** Standardized incidence ratios (SIRs) of cholangiocarcinoma in patients with choledocholithiasis according to age at the time of choledocholithiasis diagnosis

	Extrahepatic cholangiocarcinoma				Intrahepatic cholangiocarcinoma			
	Person-year	Observed event	SIR	95% CI	Person-year	Observed event	SIR	95% CI
Total	209441.22	120	12.73	10.50–15.30	209373.99	234	18.63	16.24–21.28
< 50	43947.27	5	2.53	0.82–5.91	43936.21	9	3.41	1.56–6.49
50–59	36364.24	23	14.05	8.89–21.14	36362.63	45	20.63	15.01–27.67
60–69	52481.04	39	16.51	11.71–22.64	52378.51	92	29.27	23.53–36.03
70–79	53511.86	40	16.61	11.83–22.69	53542.25	67	20.86	16.12–26.57
**≥** 80	23136.82	13	12.48	6.64–21.29	23154.41	21	15.12	9.34–23.15

## Discussion

This nationwide retrospective cohort study revealed that CCA developed in 2.64% of patients with choledocholithiasis (1.14% for ECA and 1.48% for ICA). The SIRs peaked at 70–79 and 60–69 years of age for ECA and ICA, respectively. Furthermore, the hazard of ECA was relatively high during the first 2 years, whereas that of ICA remained steady throughout the study period. This increased risk of ECA within 2 years after choledocholithiasis diagnosis could be attributed to a hidden co-existing bile duct carcinoma. Previously, Ito et al.. revealed that out of 331 endoscopic retrograde cholangiopancreatography (ERCP) cases for choledocholithiasis, 9 (2.9%) had co-existing ECA, and 2 (22%) of these cases could not be diagnosed initially during ERCP [21]. Kimura et al. also reported that ECA demonstrated stones in 7 (4.9%) of 143 patients [22]. Additionally, this increased risk of malignancy following an early period of acute inflammation is very similar to the findings on acute pancreatitis by Sadr-Azodi et al.. in 2018 and by Park et al.. in 2023, revealing undetected pre-existing pancreatic cancer, especially when acute pancreatitis was the initial presentation [15, 23]. Therefore, close follow-up should be considered during the first 2 years after endoscopic removal of choledocholithiasis, particularly in older patients, as it could be indicative of CCA. This is crucial in endemic countries of East Asia, including South Korea, where there is a relatively high incidence of CCA [24, 25].

Additional analyses were exclusively conducted with individuals diagnosed with CCA 2 years after developing choledocholithiasis to mitigate the confounding effect caused by occult co-existing carcinoma during study enrollment. The slope of the cumulative incidence graph for CCA after choledocholithiasis, wherein the slope significantly changed at the 2-year point, indicated a distinct point of discrimination (Fig. 1). Similar changes in cancer risk at 2 years were observed in pancreatic cancer after acute pancreatitis [15, 23]. Furthermore, the median survival of patients with CCA was 8 months (range: 2.8–23.3) for ICA and 16.6 months (range: 5.3–41.9) for ECA in South Korea [20]. Assuming that 2 years may be sufficient to eliminate the confounding effect of occult co-existing carcinoma, this subset analysis revealed that the SIR was significantly higher than that in the general population, thereby providing sufficient evidence to conclude that choledocholithiasis can be a risk factor for both ECA and ICA. These results are consistent with those of previous Western studies [4–6]. In 2007, Welzel et al. reported that choledocholithiasis was a significant risk factor for both ECA and ICA using the SEER-Medicare resource. The odds ratio of choledocholithiasis was 22.5 (95% CI: 16.9–30.0) for ICA and 34.0 (95% CI: 26.6–43.6) for ECA [5]. In 2017, Petrick et al. revealed choledocholithiasis as a significant risk factor for CCA [4].

The Virchow hypothesis proposed in 1863, suggests an association between chronic inflammation and malignancies, indicating that malignant transformation could occur at sites of inflammation [26]. Subsequent clinical studies have supported this idea, demonstrating that inflammatory cells can induce carcinogenic changes, leading to malignant transformation through cumulative genetic mutations [27–31]. Acute pancreatitis, as well as chronic pancreatitis, is a well-established risk factor for pancreatic cancer development [15, 23, 27–29]. Chronic biliary inflammation caused by bile duct stones can contribute to malignant transformation through multistep sequences over time, involving hyperplasia, dysplasia, adenocarcinoma in situ, and finally, invasive adenocarcinoma in the case of CCA [32–34]. Additionally, extrahepatic lithiasis may be associated with cholestasis, influencing the intrahepatic environment. Factors contributing to biliary stone development, such as altered bile composition, metabolic syndrome, or liver cirrhosis, may also increase the risk of ICA [5], which are common risk factors for both ECA and ICA [7, 35].

This study had several limitations. First, the differentiation of perihilar CCA subtypes was challenging because KCD codes (version 6) lacked specific subcodes for perihilar CCA when the HIRA database was in operation. Consequently, it could have been incorrectly recorded as either ECA or ICA. This limitation was recently addressed in the new version of the KCD codes implemented on January 1, 2021. Second, the special code (V193) for the national aid program for critical and rare diseases was not considered. Additionally, the code for ECA in KCD also included ampulla of Vater cancer, cystic duct cancer, and neuroendocrine carcinoma, even though these diseases exhibited much lower occurrences than those of CCA. Thus, the actual magnitude may be slightly lower than that observed in this study. Third, the coding system did not differentiate bile duct stones into intrahepatic and extrahepatic ones. Therefore, we could not investigate the specific association between hepatolithiasis and ICA, even though the diagnostic codes used in this study covered patients with hepatolithiasis. Fourth, while our findings highlight a significant association between choledocholithiasis and cholangiocarcinoma, the retrospective design and short delays in capturing the diagnosis time for each patient using administrative databases precludes an inference of causation. Future studies, including mechanistic investigations or prospective cohort studies, are necessary to validate and understand this link. Fifth, there remains the possibility that some cases included in the study were secondary to tumor-induced cholestasis, despite implementing a two-year washout period following the diagnosis of choledocholithiasis to mitigate this risk because the initial imaging could not be systematically reviewed in this population-based study utilizing claims data. Finally, other potential risk factors for CCA (smoking, alcohol consumption, obesity, primary sclerosing cholangitis, choledochal cysts, or hepatitis B or C infection), socioeconomic status (sex, income, or education status), and alternative treatment options for choledocholithiasis (surgical or radiological intervention), could not be explored. However, the strengths of our study lie in its population-based cohort design, encompassing nearly all patients diagnosed with choledocholithiasis from 2008 to 2009 in South Korea. The SIR was estimated by comparing them with the Korean standard population. Therefore, this population-based study, utilizing the HIRA database of South Korea, holds the potential to evaluate the association between choledocholithiasis and CCA.

In 2014, a nationwide retrospective cohort study using the Taiwan National Health Institute Research Database reported that patients with cholelithiasis have a higher risk of developing gastrointestinal cancer, particularly gallbladder and extrahepatic bile duct cancer, and among the patients with cholelithiasis, those who underwent cholecystectomy were found to have an increased risk of colorectal and stomach cancers [36]. Meanwhile, another study indicated that cholecystectomy was significantly associated with an increased risk of cholangiocarcinoma, particularly extrahepatic cholangiocarcinoma [37]. However, endoscopic sphincterotomy performed during ERCP was reported to be unlikely to be the cause of extrahepatic cholangiocarcinoma [38]. Therefore, our study results, based on patients treated with ERCP would be more reliable than those from studies involving patients treated with cholecystectomy.

## Conclusion

We observed a significantly increased risk of CCA in patients with choledocholithiasis compared to the general population. The risk particularly peaked in those in their 70s for ECA and in their 60s for ICA. Therefore, careful surveillance for CCA development should be provided to patients in whom choledocholithiasis occurs at a relatively older age.

## Electronic supplementary material

Below is the link to the electronic supplementary material.

Supplementary Material 1

## Data Availability

The study participants did not provide written consent for their data to be shared publicly. Therefore, owing to the sensitive nature of the research, supporting data is available only upon reasonable request to the Health Insurance Review and Assessment Service in South Korea (https://opendata.hira.or.kr/home.do).

## Abbreviations

CCA: Cholangiocarcinoma
CI: Confidence interval
ECA: Extrahepatic cholangiocarcinoma
ERCP: Endoscopic retrograde cholangiopancreatography
HIRA: Health Insurance Review and Assessment Service
ICA: Intrahepatic cholangiocarcinoma
KCD: The Korean Standard Classification of Disease
NHIS: The National Health Insurance Service
SIR: Standardized incidence ratio
