# Supplementary material for: Choledocholithiasis as a risk factor for cholangiocarcinoma: a nationwide retrospective cohort study
Source: BMC Gastroenterol. 2025 Mar 5;25:138. doi: 10.1186/s12876-025-03746-w (PMC11883955; doi:10.1186/s12876-025-03746-w)
Supplement: Supplementary file 1 — Supplementary Material 1 [file 12876_2025_3746_MOESM1_ESM.docx]

**Supplementary Table 1.** The codes of the Korean Standard Classification of Disease (KCD) used in this study

| Category | KCD code |  |
| --- | --- | --- |
| **Diagnosis** | **K80.50** | Calculus of bile duct without cholangitis or cholecystitis, without mention of obstruction |
|  | **K80.51** | Calculous of bile duct without cholangitis or cholecystitis, with obstruction |
|  | **K80.30** | Calculous of bile duct with cholangitis, without mention of obstruction |
|  | **K80.31** | Calculous of bile duct with cholangitis, with obstruction |
|  | **K80.40** | Calculous of bile duct with cholecystitis, without mention of obstruction |
|  | **K80.41** | Calculous of bile duct with cholecystitis, with obstruction |
|  | **C22.1** | Malignant neoplasm of intrahepatic bile duct |
|  | **C24.0** | Malignant neoplasm of extrahepatic bile duct |
| **Procedure** | **Q7761** | Endoscopic sphincterotomy |
|  | **Q7762** | Endoscopic biliary or pancreatic drainage |
|  | **Q7764** | Endoscopic removal of biliary or pancreatic stone  (Basket or balloon catheter, mechanical lithotripsy) |
|  | **Q7765** | Endoscopic removal of biliary or pancreatic stone (electrohydraulic lithotripsy) |
